# The impact of vascular margin invasion on local recurrence after pancreatoduodenectomy in pancreatic adenocarcinoma

**DOI:** 10.1007/s00423-024-03301-3

**Published:** 2024-04-12

**Authors:** Julio Cuesta López, Benedetto Ielpo, Mar Iglesias, Fernando Burdío Pinilla, Patricia Sánchez-Velázquez

**Affiliations:** 1 Pompeu-Fabra University, Barcelona, Spain; 2 Department of Surgery, Division of Hepato-Biliary and Pancreatic Surgery, University Hospital del Mar-IMIM (Hospital del Mar Medical Research Institute), Universitat Pompeu Fabra, 08003 Barcelona, Spain; 3 Department of Pathology, University Hospital del Mar-IMIM (Hospital del Mar Medical Research Institute), Universitat Pompeu-Fabra, Barcelona, Spain

**Keywords:** Margin, Pancreatoduodenectomy, Pancreatic adenocarcinoma, Whipple

## Abstract

**Purpose:**

Pancreatic ductal adenocarcinoma (PADC) still has nowadays a very impaired long-term survival. Most studies are focused on overall survival; however, local recurrence occurs about up to 50% of cases and seems to be highly related with margin resection status. We aim to analyze the impact of vascular resection margins on local recurrence (LR) and to assess its impact on overall and disease-free survival.

**Methods:**

Eighty out of 191 patients who underwent pancreatoduodenectomy in a university hospital between 2006 and 2021 with PDAC diagnosis were analyzed and vascular margin status specifically addressed. Univariate and multivariate were performed. Time to LR was compared by using the Kaplan–Meier method and prognostic factors assessed using Cox regression hazards model.

**Results:**

LR appeared in 10 (50%) of the overall R1 resections in the venous margin and 9 (60%) in the arterial one. Time to LR was significantly shorter when any margin was overall affected (23.2 vs 44.7 months, *p* = 0.01) and specifically in the arterial margin involvement (13.7 vs 32.1 months, *p* = 0.009). Overall R1 resections (HR 2.61, *p* = 0.013) and a positive arterial margin (HR 2.84, *p* = 0.012) were associated with local recurrence on univariate analysis, whereas arterial positive margin remained significant on multivariate analysis (HR 2.70, *p* = 0.031).

**Conclusions:**

Arterial margin invasion is correlated in our cohort with local recurrence. Given the limited ability to modify this margin intraoperatively, preoperative therapies should be considered to improve local margin clearance.

## Introduction

Pancreatic ductal adenocarcinoma (PDAC) is currently one of the most lethal cancers in oncology due to its early local invasion capacity with a 5-year relative survival rate of 8% [1, 2] and it is expected to be indeed the second leading cause of cancer-related death in 2030 [3–6].

Among multiple clinical variables, resection margin status seems to be one of the most important factors enrolled in the prognosis [7–9]; however, there was a lack of consensus regarding the definition of an adequate margin of resection before the publication of Verbeke’s protocol [10, 11]. This milestone study revolutionized the assessment of R0 (> 1 mm) and R1 (< 1 mm) status, and therefore, R0 resection rates in the literature vary between 15 and 83% depending on the applied protocol which precludes data comparison [12]. Along with the previous, the definition of resectability of PADC has experienced a constant evolution in the last two decades starting with the introduction of the concept borderline resectable PADC (BR-PADC) in 2006 by the NCCN, and it is merely a subrogate indicator of the likelihood to achieve an R0 margin status.

Despite advances in systemic therapies such as targeted chemotherapy, surgical resection together with targeted chemotherapy is currently the only possible curative treatment [13]. In the last decades, it has been advocated that neoadjuvant treatment might have a benefit effect on local control reducing the rate of positive resection margins [14]; however, further investigation is needed to assess the impact of pre-operative chemotherapy on overall survival (OS) and disease-free survival (DFS) [15] as data is still controversial.

Due to the aggressive nature of the tumor, even after curative-intent resection, patients experience postoperative recurrence which varies from 50 to 90% [16, 17]. Although many of them will experience systemic recurrence in their lifetime, local recurrence (LR) constitutes an important part of this recurrence estimated to be as high as 50% and it is not specifically addressed in many studies [18, 19]. The structures mainly involved in LR are the hepatoduodenal ligament, the common hepatic artery, remnant pancreas, the superior mesenteric artery (SMA), and the superior mesenteric vein (SMV). Positive resection margins, especially SMA margin, are usually associated with local recurrence and impaired survival [20].

The main object of this study is to evaluate microscopic involvement on at least one of the inked vascular resection margins and assess its impact on local recurrence and to identify factors correlated with resection margin involvement.

## Material and methods

### Study design and data collection

A prospectively maintained database was queried for all patients who underwent PD between January 2006 and December 2021 and had a final pathologic diagnosis of PDAC at Hospital del Mar. Ethical approval was obtained from the Institutional Review Board at University Hospital del Mar (num. 2022/10653) and the study was performed in accordance with the guidelines indicated by Good Clinical Research Practices and the Declaration of Helsinki. Furthermore, the confidentiality of patient data is respected, in compliance with the European Data Protection Regulation (EU) 2016/679 and Organic Law 3/2018, of December 5, on the Protection of Personal Data and guarantee of the digital rights. Human ethics and consent to participate declarations are not applicable.

All consecutive patients that underwent PD (non-pylorus preserving panreatoduodenectomy) either those that received neoadjuvant treatment or upfront surgery and regardless the approach, open or laparoscopic, were included in this study. Patients who presented a pancreas head tumor no adenocarcinoma like neuroendocrine tumors or in absence of informed consent were excluded. The selection of patients who were candidates for chemotherapy was based on the recommendations that were valid for each period. So, neoadjuvant chemotherapy was used in borderline or locally advanced PDAC [21], whereas adjuvant chemotherapy consisting in gemcitabine + nab-paclitaxel or FOLFIRINOX was administered in all PDAC, unless there was any contraindication.

Baseline characteristics were extracted from the prospective clinical database and collected by two independent investigators. All operative procedures have been performed by three experienced pancreatic surgeons who have reached their learning curve. All patients received at least 1 month postoperative computed tomography to rule out complications, and hereafter followed the normal oncological surveillance every 3 months the first 2 years and every 6 months the following till complete 5 years.

### Definition of primary and secondary end-points

The primary end-point was to analyze weather the SMV margin and the SMA margin (measured in mm) in Whipple’s procedure had an impact on the local recurrence (LR). Local recurrence was defined as the appearance of new mass lesions by contrast-enhanced computed tomography (CT), magnetic resonance imaging, or positron emission tomography-CT within the resection field, pancreatojejunal anastomosis, remnant pancreas, or retroperitoneal site outside of the surgical bed [22].

Secondary end-points were included to evaluate overall survival (OS) and disease-free survival (DFS). OS is defined as the time from the date of resection to the date of death from any cause or last known follow-up. DFS will be measured from the date of resection until the date of recurrence diagnosis [23].

### Histopathological evaluation of resection specimens

Tumors were staged according to the 8th AJCC TNM classification. Histological report of diagnosed with a PADC previous to the 8th edition was reviewed and adapted to unify criteria. PD specimens were serially sliced in a perpendicular plane to the duodenal axis according to the Leeds Pathology Protocol [10, 24]. Margin status was determined for the transection margins (pancreatic neck, proximal and distal enteric margins, and common bile duct), as well as for the circumferential resection margins (posterior, i.e., superior mesenteric artery (SMA), and medial, i.e., superior mesenteric vein/portal vein (SMV/PV) margins). The results of the standard histopathological analysis report were reviewed by the investigators.

### Statistical methodology

The Kolmogorov–Smirnov test was used to check the normality of the data and the Levene test for equality of variances. Continuous variables were expressed as median and interquartile range (IQR). Categorical variables were expressed as absolute numbers and percentages. Kaplan–Meier curves were used to estimate unadjusted median LR, DFS, and OS and their 95% confidence intervals (CI), and compared using the log-rank test. Multivariable Cox-proportional hazard analysis was performed to assess the association between resection margin status and LR, both DFS and OS and adjusted for potential confounders including age, gender, Eastern Cooperation Oncology Group (ECOG) performance score, differentiation tumor grade, TNM stage, and neo-adjuvant chemotherapy. All statistical analyses were performed using the software IBM SPSS Statistics version 28.0 (IBM, Armonk, NY, USA) [25].

## Results

**Fig. 1:**
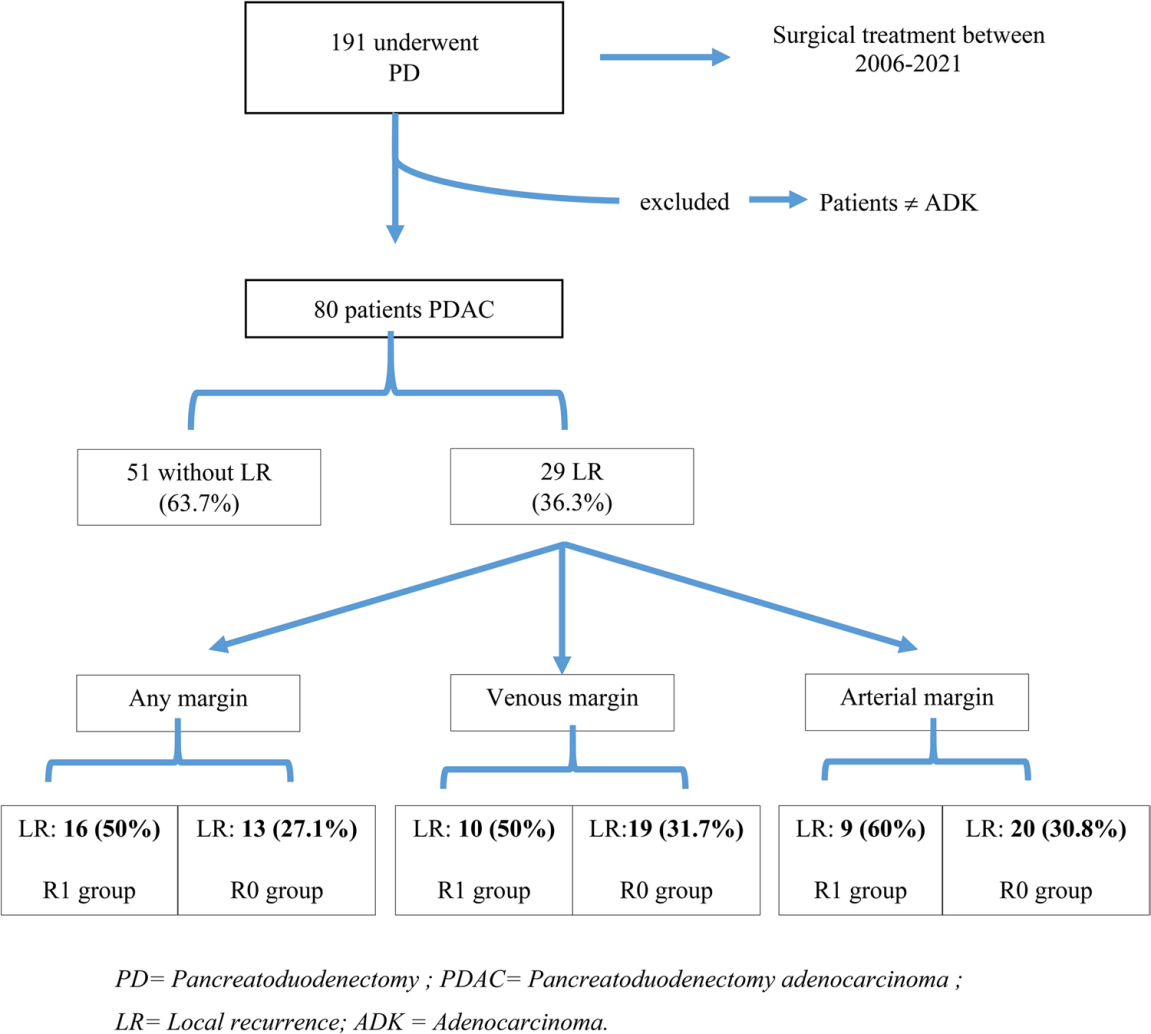
Study flow-chart

Between 2006 and 2021, there were 191 patients who underwent surgical PD in Hospital del Mar. After applying the exclusion criteria, 80 patients were finally eligible for inclusion in this study (Fig. 1). Demographic, clinical, and pathologic characteristics of the whole cohort are depicted in Table 1.

**Table 1:** Demographic and clinicopathological features of the 80 patients with PDAC

Variable	Patients with PDAC (*n* = 80)
Age (years), median (range)	67 (36–91)
Gender (male), *n* (%)	49 (61.3%)
Performance status, *n* (%)	
0	42 (52.5%)
1	25 (31.3%)
2	3 (3.8%)
3	0 (0%)
4	1 (1.3%)
Not reported	9 (11.3%)
ASA classification, *n* (%)	
1	2 (2.5%)
2	40 (50%)
3	36 (45%)
4	1 (1.3%)
Not reported	1 (1.3%)
Tumor size, *n* (%)	
T1	6 (7.5%)
T2	16 (20%)
T3	51 (63.7%)
T4	5 (6.3%)
Not reported	2 (2.6%)
Nodal metastasis, *n* (%)	
N0	26 (32.5%)
N1	31 (38.8%)
N2	23 (28.7%)
Not reported	0 (0%)
Distant metastasis, *n* (%)	
M0	62 (77.5%)
M1	13 (16.2%)
Not reported	5 (6.3%)
STAGE, *n* (%)	
I	10 (12.5%)
II	44 (55%)
III	26 (32.5%)
Not reported	0 (0%)
Neoadjuvant QT	7 (8.8%)
Adjuvant QT	48 (60%)

*QT *chemotherapy

**Fig. 2:**
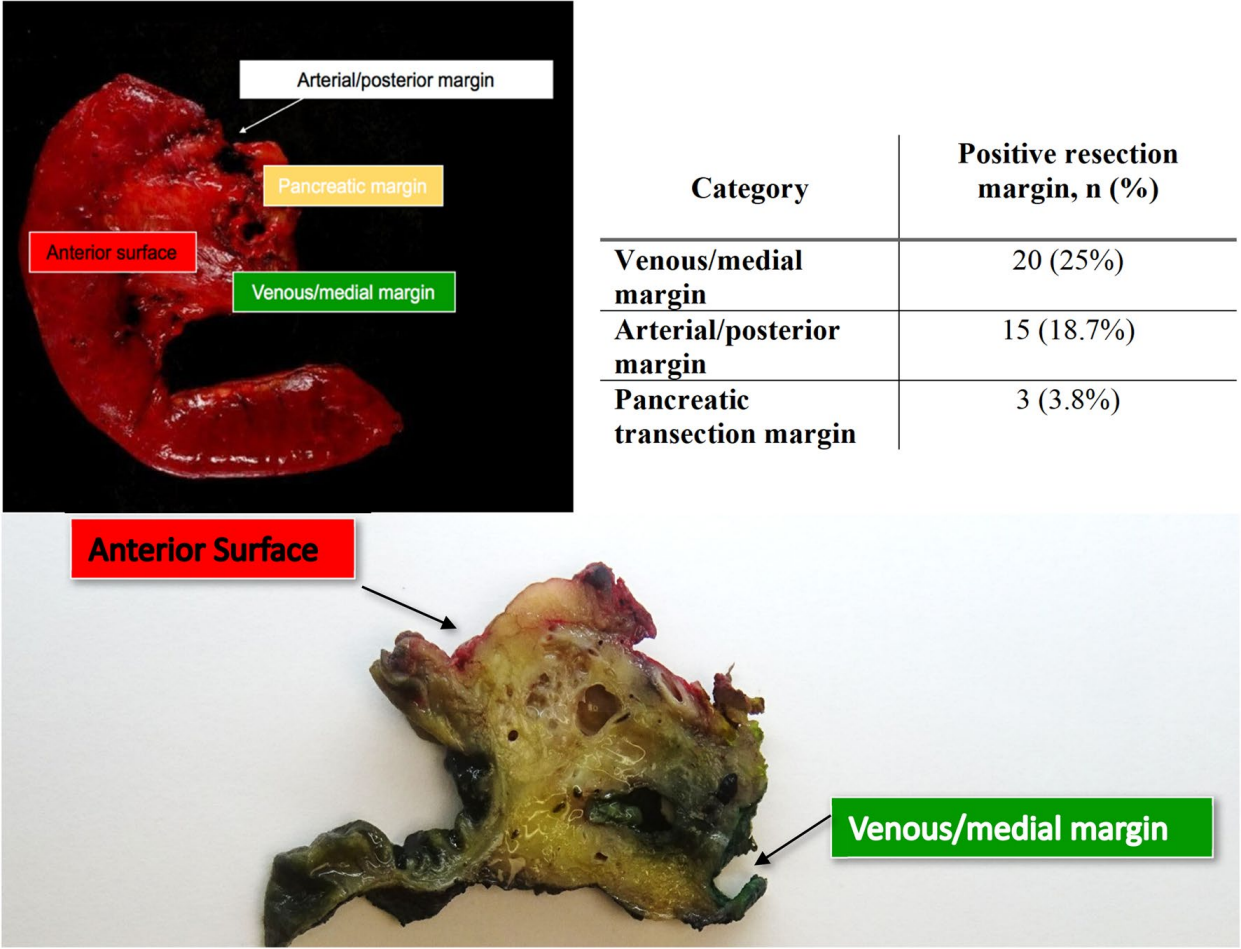
Resection margins involved in PD and anatomopathological pancreas specimen

Concerning the R status, R0 resection was achieved in 48 patients (60%) whereas R1 in 32 patients (40%). A positive venous margin was the most common site of margin positivity followed by a positive arterial margin (25 vs 18.75% respectively). The pancreatic transection margin was positive in 3 patients (3.8%) (Fig. 2).

### Primary end-point

Out of the 80 patients finally included in this study, 29 patients (36.3%) presented a LR. Within this group, there were more patients with LR in those who presented an R1 status 50% compared to those with a R0 status 27.1% regardless the affected margin. When specifically assessing the LR split by resection margin, we found that in the venous margin 10 patients suffered a LR from the overall R1 resections and 19 from the overall R0 resections (50% and 31.7% respectively). Similarly, while in the arterial margin, 9 patients from the R1 group experienced a LR and 20 from the R0 resection group (60% and 30.8% respectively) (Fig. 1). Median follow-up was 50 months.

**Fig. 3:**
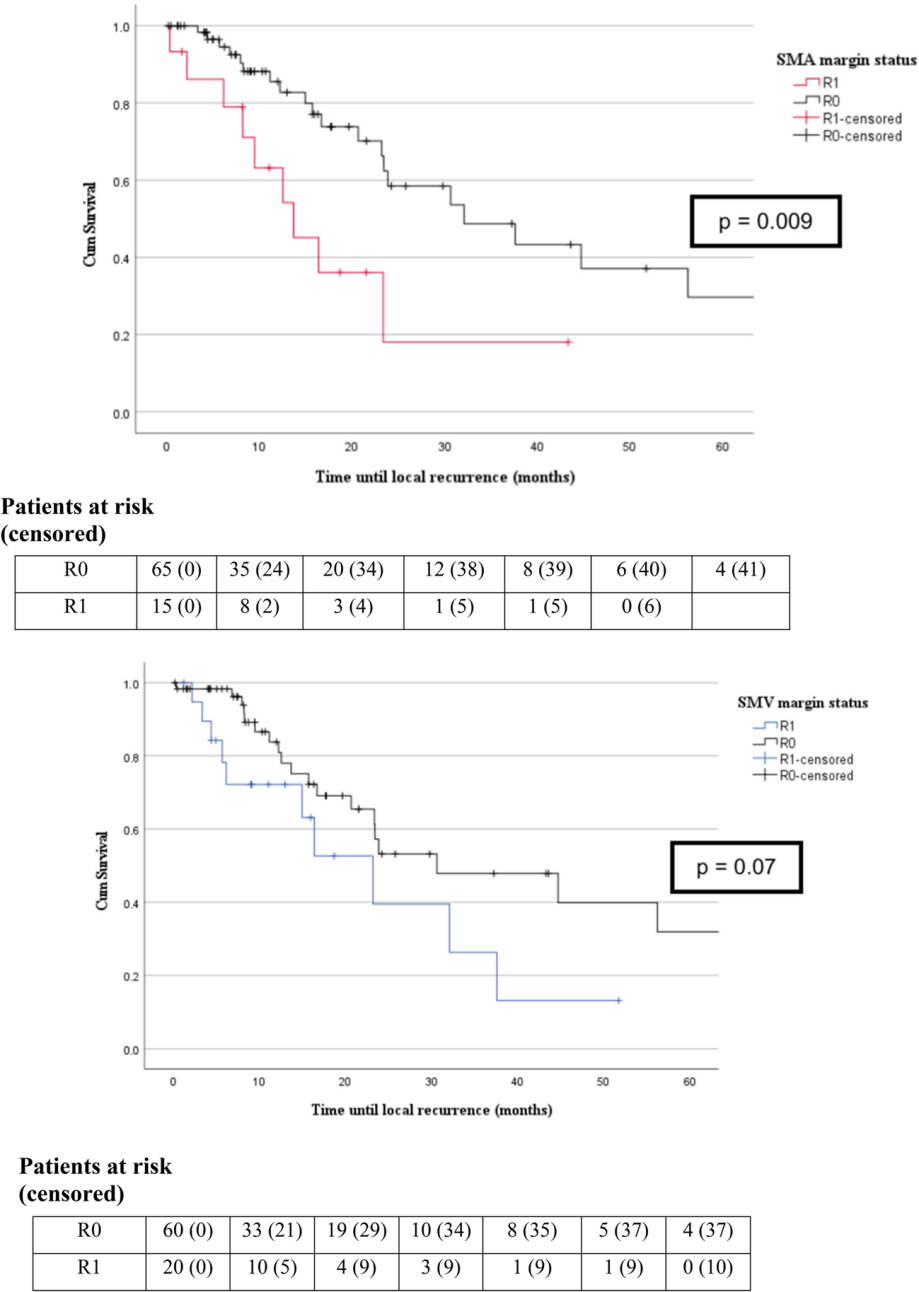
Kaplan–Meier survival curves for arterial and venous margin

Time to LR was significantly shorter in the R1 group regardless the affected margin (23.2 [95% CI 9–37.4] vs 44.7 months [95% CI 19.8–69.6], *p* = 0.01). Whereas the affected venous margin was not a significant factor for LR (23.2 [95% CI 12.6–33.8] vs 30.6 months [95% CI 7.5–53.8], *p* = 0.07), the arterial margin had a significant impact (13.7 [95% CI 6.5–20.9] vs 32.1 months [95% CI 14.9–49.3], *p* = 0.009) (Fig. 3).

On univariate survival analysis, an overall R1 resection was associated with decreased disease-free local recurrence (HR 2.615 (CI 1.226–2.576), *p* = 0.013) and also was the arterial margin (HR 2.840 (1.259–6.406), *p* = 0.012). Venous margin and other variables were not found to be statistically significant (Table 2).

**Table 2:** Univariate and multivariate Cox regression analysis of predictive factors associated with local recurrence

	Univariate analysis	Multivariate analysis
	Local recurrence HR (95% CI) *p*-value	Local recurrence HR (95% CI) *p*-value
Resection margin (R1)	**2.615 (1.226–2.576) 0.013**	
Arterial margin (R1)	**2.840 (1.259–6.406) 0.012**	**2.703 (1.094–6.683) 0.031**
Vascular margin (R1)	2.021 (0.930–4.391) 0.076	0.500 (0.224–1.114) 0.090
Neoadjuvant chemotherapy	0.763 (0.228–2.556) 0.661	0.712 (0.179–2.832) 0.630
Differentiation grade	1.088 (0.670–1.768) 0.734	1.295 (0.734–2.285) 0.372
Tumor size	1.169 (0.881–1.551) 0.279	1.135 (0.839–1.534) 0.412
N status	1.440 (0.901–2.301) 0.127	1.467 (0.843–2.553) 0.175
Age (> 75)	0.623 (0.293–1.326) 0.219	1.806 (0.822–3.967) 0.141

The bold values are the statistically significant (*p*) values

*HR* hazard ratio; *CI* confidence interval

Multivariate analysis model was constructed including other factors that may impact on survival, such as neoadjuvant chemotherapy, tumor differentiation, maximum tumor size (measured on the log scale), N status, age (> 75), and involvement of vascular and arterial margin. Only the arterial margin was found to be an independent predictor of local recurrence (HR 2.703 (1.094–6.683), *p* = 0.031). Further covariates such as overall resection margin were excluded as were determined based on the addition of vascular and arterial margins. These results are summarized in Table 2.

### Secondary end-points

The median OS of the whole cohort was of 24.48 months (95% CI 14.86–40.93). Patients with a R0 resection survived 24.4 months (95% CI 14.1–34.7) compared to patients with R1 resection 28.4 months (19.8–36.9), *p* = 0.963. When specifically addressed the surgical margins, patients with R0 venous margin have not experienced increased OS when compared to patients with R1 resection (26.2 [95% CI 19.6–32.7] vs 27 months [95% CI 1.7–52.2] respectively, *p* = 0.989). Neither patients with an arterial R0 resection have experienced increased OS when compared to patients with R1 resection (24.5 [95% CI 20.4–28.7] vs 34.1 [17.9–50.3] months respectively, *p* = 0.577).

The median DFS of the whole cohort was 11.12 months (95% CI 5.82–22.79). Patients with an overall R0 resection have experienced increased DFS when compared to patients with R1 resection, but not significant (15.7 [95% CI 9.2–22.3] vs 13.7 months [95% CI 8–19.4] respectively, *p* = 0.502). When specifically addressed the surgical margins, patients with R0 venous margin have not experienced increased DFS when compared to patients with R1 resection (15.7 [95% CI 10.7–20.8] vs 15 months [6.4–23.5] respectively, *p* = 0.488). Patients with an arterial R0 resection have experienced increased DFS when compared to patients with R1 resection, but not significant (15.7 [95% CI 10.6–20.8] vs 12.6 [95% CI 8–17.1] respectively, *p* = 0.496).

## Discussion

In this study, we investigated the relationship between the positive surgical margin and local recurrence after PD. Our results showed that LR appeared more frequently in patients with an R1 resection, particularly in patients with a positive arterial margin, and was the only independent factor related with local recurrence on univariate and multivariate analysis. This can be explained by the proximity of the tumor to the perineural plexus surrounding the superior mesenteric artery and the inability to resect additional tissue when the surgeon is confronted with a positive margin along the artery. Interestingly, other factors which potentially could have impact on LR such as neoadjuvant chemotherapy, differentiation, tumor size, and N status have failed in our cohort to demonstrate a relationship with LR.

Multiple studies go along with our results particularly with relation to R1 arterial resection [8, 26, 27] while the venous margin, which did not have significant impact on local recurrence in our cohort, might have been underpowered due to the small sample size [28]. It seems logical to assume this relationship, but in a recent review was reported that less of 50% of the studies reported the R-status sorted by anatomical margins and very few assessed specifically LR stratified by arterial or venous margin.

In terms of overall survival and disease-free survival, we found no impact of R1 resections similarly to Sugiura [27] and Gebauer et al. [29]; however, large study cohorts such as Ghaneh et al. [8] and Strobel et al. [30] clearly correlated a R1 resection with both. This large variety and disparity results might be related to the different criteria employed to determine the cut-off point of R1 resection worldwide, as in the USA; generally, the criteria of R1 definition for > 0 mm from the surgical edge is still followed. Moreover, Gebauer et al. [29] and Chang et al. [31] have shown that incrementing the cutoff point to > 1.5 mm also significantly improves the overall survival.

These findings have an important clinical relevance, since nowadays several strategies could be applied for reducing the risk of R1 resections, and subsequently, achieving better outcomes. Initial treatment with neoadjuvant chemotherapy has shown the potential to downsize the tumor and decrease the rate of R1 resections [15, 32]. Recently, Truty et al. [33] achieved 94% of R0 margins with a combination of Folfirinox/Gemcitabine with Nab-paclitaxel in neoadjuvant basis. In our study, neoadjuvant therapy (NAT) has not been significant for local recurrence, but this is perhaps limited by the small number of patients who received neoadjuvant therapy which can be considered a limitation of the study. The initial outcomes of the multicenter randomized PREOPANC trial carried out by Versteijne et al. [34] comparing NAT to upfront surgery showed significant higher rates of R0 resection, lower local recurrence rates, but not positive results for overall survival. However, in the recent published PREOPANC long-term outcomes [35], the 5-year OS rate was significantly higher in the neoadjuvant group. As neoadjuvant therapy has shown promising results for resectable and borderline resectable PDAC to increase R0 resections, decrease the risk of local recurrence, and enlarge overall survival compared to up-front surgery, it should be considered as a possible strategy for PDAC with expected narrow margins.

“Artery first approach” (AFA) is a surgical technique characterized by a meticulous clearance of the superior mesenteric artery to achieve a higher rate of R0 and theoretically it could influence LR [36]. Whereas a metanalysis by Ironside et al. [37] demonstrated significant differences in terms of R0 resection and local recurrence, a recent multicenter randomized controlled trial by Sabater et al. [38] has concluded that AFA has neither impact on the rates of affected margins nor influence on local recurrence rate. Other surgical options such as TRIANGLE [39, 40] operation and arterial divestment which have been recently described could also be a tool in the surgeon armamentarium to increase R0 resections.

Regarding adjuvant treatment, patients with high-risk pathological features such as R1 resection in the definitive report or nodal involvement and adjuvant radiotherapy (RT) might increase survival [39, 40]. Moreover, Kamarajah et al. [39] have proven that in R0 resection but with node-positive disease, the use of radiotherapy increases survival in 2–4 months in comparison to only chemotherapy. However, these studies, in favor of adjuvant RT, have low scientific evidence; therefore, its use is not yet broadly stablished in PDAC treatment protocols.

There are, however, some limitations to this study. First, this was a retrospective study design despite the prospectively collected data. Second, the number of patients with local recurrence was quite small, so a multicenter study with a bigger cohort could show stronger results. Third, data was obtained from patients undergoing surgery since 2006, so changes to treatment strategies and chemotherapy regimens have appeared throughout the study. Fourth, although we adjusted our analyses for several potential confounders, the possibility of residual and unmeasured confounding remains.

In conclusion, local recurrence after PDAC is common and it seems to be related with the affected margin, specially the arterial one due to the inability to clear surgical margins. Developing new strategies to reduce margin invasion, pre- and postoperative therapies could improve local recurrence rates and therefore better survival.

## Data Availability

All data supporting the findings of this study are available upon reasonable request.
